# Intricate Transcriptional Networks of Classical Brown and Beige Fat Cells

**DOI:** 10.3389/fendo.2015.00124

**Published:** 2015-08-12

**Authors:** Jun Hong Park, Wonhee Hur, Sean Bong Lee

**Affiliations:** ^1^Department of Pathology and Laboratory Medicine, Tulane University School of Medicine, New Orleans, LA, USA

**Keywords:** classical brown fat, beige, brite, transcriptional regulation, transcription factors, miRNAs, lincRNAs

## Abstract

Brown adipocytes are a specialized cell type that is critical for adaptive thermogenesis, energy homeostasis, and metabolism. In response to cold, both classical brown fat and the newly identified “beige” or “brite” cells are activated by β-adrenergic signaling and catabolize stored lipids and carbohydrates to produce heat via UCP1. Once thought to be non-existent in adults, recent studies have discovered active classical brown and beige fat cells in humans, thus reinvigorating interest in brown and beige adipocytes. This review will focus on the newly discovered transcription factors and microRNAs that specify and orchestrate the classical brown and beige fat cell development.

## Two Types of Thermogenic Cells: Classical Brown and Beige Fat Cells

Non-shivering thermogenesis in mammals is carried out by a group of specialized fat cells known as brown adipocytes. Classical brown adipocytes are generated during embryogenesis in distinct brown adipose tissue (BAT) depots, such as the axillary, interscapular, and subscapular regions (1, 2). Classical BAT is abundant in rodents and hibernating mammals and it functions to maintain their body temperature in cold climate. Human infants are also born with classical BAT but it disappears over time and was considered to be non-existent in adults (3). However, PET-CT imaging studies with ^18^F-fluorodeoxyglucose have discovered active BAT in the neck and supraclavicular regions in adult (4–8). These findings have revitalized the research on BAT and the efforts to utilize it as a potential therapy against obesity and other metabolic diseases.

Recent studies in rodents and humans have discovered a second type of brown fat cells known as the beige or brite (brown in white) cells (2, 9). Beige cells are generated postnatally within white adipose tissues (WAT) in response to cold or adrenergic stimulation. Both classical brown fat and beige cells are rich in mitochondria and uniquely express uncoupling protein 1 (UCP1), an inner mitochondria membrane protein that produces heat by uncoupling the proton gradient from ATP synthase. Although both brown and beige cells share the same thermogenic function, they arise from entirely different cell lineages (2, 10). Classical brown fat cells arise from myogenic progenitors that express Myf5 and Pax7 myogenic transcription factors (11, 12) in specific BAT depots during development. In contrast, beige cells are made postnatally in WAT depots and arise from Myf5-precursors that express platelet-derived growth factor receptor α (PDGFRα) (10, 13–15) or through transdifferentiation of mature white adipocytes (16–18) in response to cold or β-adrenergic stimulation. A recent study has also shown that beige cells can arise from smooth muscle cell (Myh11+) progenitors (19). Several mouse genetic lineage-tracing studies have led to discordant results regarding how beige cells are generated (13, 15, 17–19). The studies that permanently marked mature white adipocytes in subcutaneous (subQ) WAT showed that cold-induced beige cells are derived from mature white adipocytes in subQ WAT (17, 18), while another study showed that some beige cells are generated *de novo* from progenitors (15). In contrast, studies that genetically marked PDGFRα+ progenitors in epididymal WAT (13) or smooth muscle progenitors (Myh11+) in subQ WAT (19) demonstrated that beige cells are derived from the respective progenitors. While further studies are needed to clarify these issues, these studies clearly demonstrate that different WAT depots have different “browning” capacity and might employ different mechanisms to generate beige cells.

The discovery of active BAT in humans has raised the issue of whether human BATs comprises classical brown or beige fat cells. Several studies have revealed that adult human BAT is more similar to the mouse beige cells (10, 20–22), while other studies showed that it is closer to the classical BAT (23, 24). Hence, similar to rodents, it is likely that adult humans possess both classical brown and beige fat cells, depending on different anatomical locations.

## Preservation of Core Transcriptional Hierarchy in Brown and White Adipogenesis

**Figure 1:**
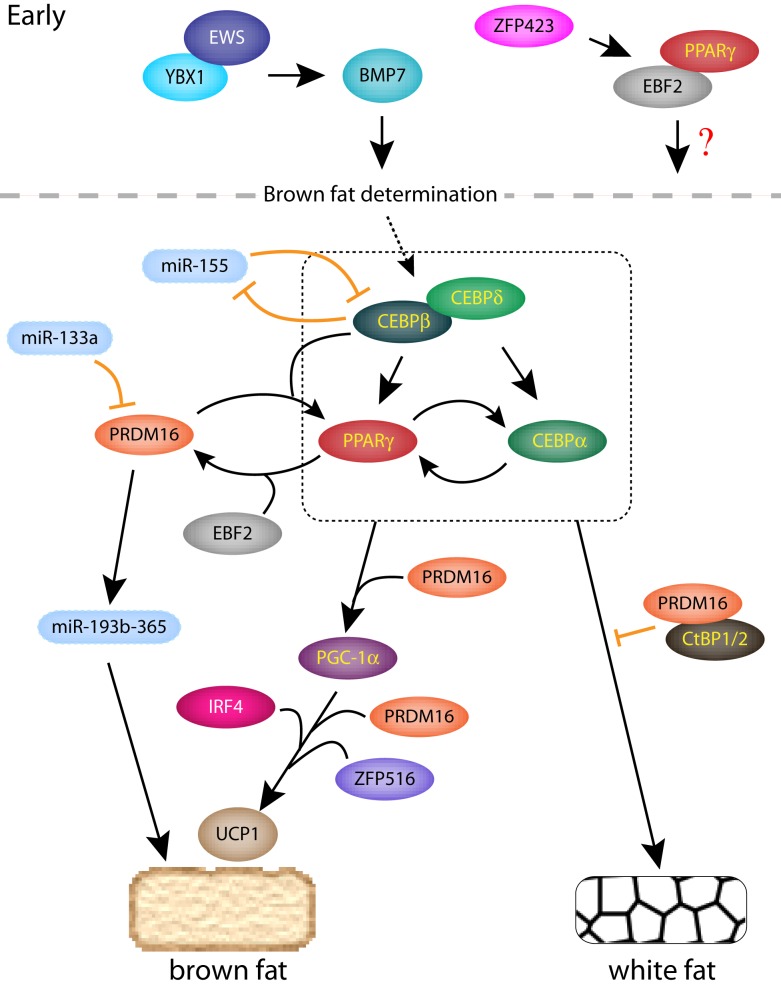
**A regulatory network of transcription factors and miRNAs in classical brown and white adipogenesis**. Early transcription factors are shown above the dotted line and the box indicates the core transcription factors critical for brown and white adipogenesis.

During adipogenesis, external adipogenic signals activate a cascade of core transcription factors that are critical for both brown and white adipocyte differentiation. The sequential activation of these transcription factors has been elegantly worked out in 3T3-L1 cells (25), which were first established in Howard Green’s lab from Swiss albino mouse embryonic fibroblasts (MEFs) (26) using a 3T3 protocol (27). Adipogenic stimulation of either white or brown preadipocytes leads to a sequential activation of core transcription factors (25, 28, 29) (Figure 1). One of the earliest activated transcription factors are CCAAT-enhancer-binding protein-β (CEBP-β) and CEBP-δ, which then form a heterodimer and transcriptionally activate peroxisome proliferator-activated receptor γ (PPARγ), along with another family member, CEBP-α. PPARγ is a member of the nuclear hormone receptor superfamily and is the master regulator of adipogenesis as its sole expression is sufficient to convert fibroblasts into adipocytes (30). Upon activation, PPARγ activates the transcription of CEBP-α and many other genes involved in fatty acid synthesis, lipid storage, and glucose metabolism (25). CEBP-α then reciprocally activates PPARγ as well as other adipogenic genes. While PPARγ and the CEBP-family proteins are the core transcriptional regulators of both BAT and WAT adipogenesis, auxiliary transcription factors, such as Kruppel-like factor 5 and 15 (KLF5 and KLF15), also modulate general adipogenesis (25). Interestingly, Zfp423, which contains 30 Kruppel-like zinc fingers and a SMAD-binding domain, was identified as a regulator of preadipocyte determination by activating the transcription of PPARγ (31) (Figure 1). Deletion of *Zfp423* in mice inhibits both brown and white adipogenesis.

## Transcriptional Regulators of Brown and Beige Fat Cells

Several recent studies have revealed brown and beige fat-specific transcriptional regulators as well as microRNAs (miRNAs) and long intergenic non-coding RNAs (lincRNAs), which will be described in detail.

## *P*RD1-BF-1-*R*IZ1 Homologous-*D*omain *M*ember 16

*P*RD1-BF-1-*R*IZ1 homologous-*D*omain *M*ember 16 (PRDM16) and its close homolog PRDM3 were first identified as the histone 3-Lys 9-monomethyltransferases (H3K9me1) that are critical for heterochromatin organization (32). By examining transcription-related genes enriched in brown fat versus white fat, Seale et al. identified PRDM16 as a brown fat-specific transcription factor (33). Ectopic expression of PRDM16 in WAT results in increased beige cell formation in the mouse. Conversely, knockdown of PRDM16 blocks brown fat differentiation. Remarkably, knockdown of PRDM16 in primary brown preadipocytes leads to myocyte differentiation and ectopic PRDM16 expression in myoblasts turns them into brown fat cells upon adipogenic stimulation (11). These results suggest that PRDM16 controls a cell fate switch between brown fat and myocyte differentiation in bipotent progenitors. Interestingly, another H3K9 methyltransferase, EHMT1, interacts with PRDM16 and is required for BAT development (34). PRDM16 forms a complex with CEBPβ and together, these two factors are able to convert a naïve fibroblasts or myoblasts into brown fat cells (35). Additionally, PRDM16 interacts with C-terminal binding proteins, CtBP1 and CtBP2, and represses white fat gene expression program (36), but this interaction can be displaced by PGC-1α or PGC-1β, which induces brown fat program upon binding to PRDM16 (33, 36). PRDM16 also interacts with PPARγ and enhances its transcriptional activity (11). In addition to cold and β3-agonists, PPARγ agonists can also induce beige cell differentiation in the mouse, which was shown to require PRDM16 (37). Addition of PPARγ agonists stabilizes PRDM16, likely through its interaction with PPARγ.

**Figure 2:**
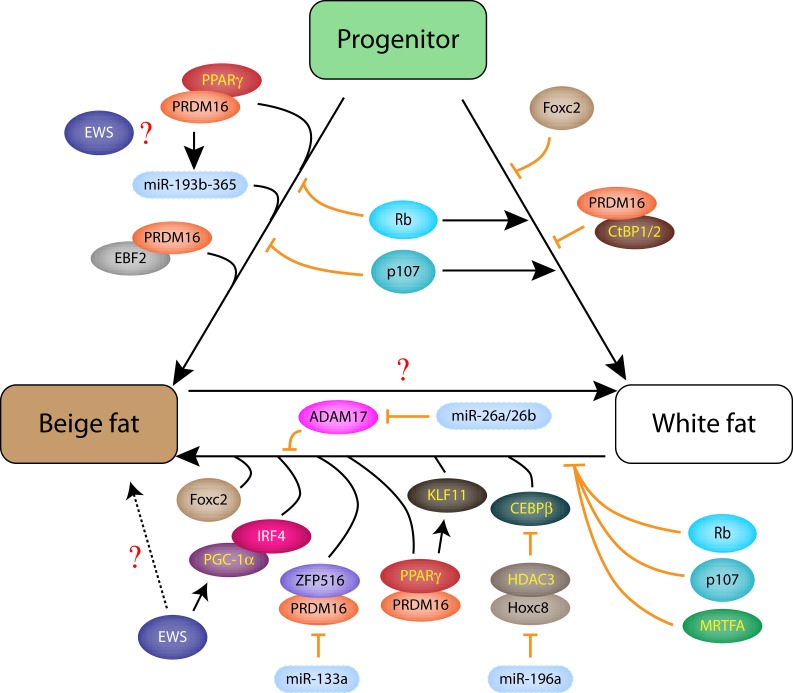
**Intricate networks of transcription factors and miRNAs in beige and white fat differentiation from progenitors or through transdifferentiation**.

Initial description of global PRDM16 knockout (KO) mice, which was postnatal lethal, reported abnormal BAT morphology with reduced brown fat gene expression and ectopic myogenic gene expression (11). A recent study showed that specific deletion of PRDM16 in postnatal adipose tissues (BAT and WAT) using Adiponectin-Cre blocks cold- or β3-agonist-induced browning of subQ WAT, but has minimal effects on classical BAT and visceral (Vis) WAT (38), demonstrating that PRDM16 is essential for beige fat formation in subQ WAT (Figure 2). Furthermore, loss of PRDM16 induces subQ WAT to adopt Vis WAT gene expression profile and reduces its thermogenic capacity. These findings indicate that while PRDM16 is required during early brown cell fate determination (according to earlier studies), it is dispensable for mature BAT thermogenesis. Therefore, it was quite surprising when a specific deletion of PRDM16 in early myogenic progenitors using Myf5-Cre showed normal BAT development (39). In contrast to the Cohen et al. study which reported minimal effects on BAT (38), adult BATs derived from Myf5-specific deletion of PRDM16 shows increased white fat differentiation and reduced thermogenesis in aged animals, suggesting a role of PRDM16 in maintaining mature BAT function (39). A simultaneous deletion of both PRDM16 and its homolog PRDM3 shows much earlier and more prominent brown fat defect than the single PRDM16-KO, although the embryonic and early postnatal (2 weeks) BAT development are minimally affected. These studies show that in the absence of PRDM16, PRDM3 can serve a compensatory role.

## FOXC2

A role of a winged helix/forkhead gene, *Foxc2*, in browning of white fat was demonstrated well before beige cell was recognized as a distinct cell type (40). Expression of *Foxc2* is highly restricted to both BAT and WAT, and adipose-specific expression of *Foxc2* using *aP2* (Fabp4) promoter in mice results in browning of WAT and hypertrophic BAT. Furthermore, Foxc2 transgenic mice are resistant to high-fat diet (HFD)-induced obesity and insulin and glucose resistance. This is at least partly due to increased mitochondria number and respiration of beige cells in WAT of transgenic mice. Intriguingly, expression of Foxc2 in 3T3-L1 cells blocks white fat differentiation by inhibiting the expression of certain PPARγ target genes (41).

## EWS

*Ewing sarcoma break point region 1* (*EWSR1*, herein termed *EWS*) encodes a highly abundant, multifunctional RNA/ssDNA binding protein (42). Originally presumed to play housekeeping roles in basic transcription and RNA splicing (43), generation of EWS-KO mouse and other studies have revealed a surprisingly diverse role of EWS in meiosis, B-cell development, prevention of cellular senescence, mitosis, DNA damage-induced alternative splicing, and miRNA regulation (44–49). More recently, it was discovered that classical BAT development was completed blocked in EWS-KO (50). Deletion of EWS results in a complete block in early embryonic classical BAT development and loss of brown fat differentiation in preadipocytes. As a member of TGF-β superfamily, BMP7, bone morphogenic protein 7, plays a critical role in the commitment of early mesenchymal progenitors to brown fat (51). In the absence of EWS, BMP7 expression is lost in embryonic BAT and in brown preadipocytes undergoing adipogenesis (50). Following adipogenic stimulation, EWS forms a complex with Y box-binding protein 1 (YBX1) and activates BMP7 transcription. Depletion of YBX1 also results in loss of BMP7 expression and a block in brown fat differentiation. Notably, loss of EWS leads to ectopic myogenic expression in EWS-KO BAT, consistent with the idea that EWS determines the classical brown cell fate. Interestingly, EWS heterozygous mice show reduced beige cell recruitment in inguinal WAT in response to PPARγ agonist or β3-adrenergic stimulation (50). However, the definitive role of EWS in beige cell development will require further studies. As both brown fat and beige cells are rich in mitochondria, it is intriguing to note that EWS was recently shown to regulate mitochondria density and function by controlling PGC-1α protein stability (52). Finally, EWS may also have a role in white adipogenesis, at least *in vitro* (53).

## EBF2

A search for brown fat-specific PPARγ-regulated promoters by ChIP-seq analysis identified an enrichment of early B-cell factor (EBF) binding sites in the PPARγ occupied DNA regions (54). It was subsequently shown that EBF2, one of the four EBF isoforms, is highly expressed in BAT compared to WAT or beige cells. Ectopic expression of EBF2 in C2C12 myoblasts or in stromal vascular fraction (SVF, which is known to contain adipocyte progenitors) leads to a strong induction of brown fat differentiation, while depletion of EBF2 blocks differentiation in brown preadipocytes. EBF2 recruits PPARγ to the PRDM16 promoter/enhancer region and synergistically activates its expression. EBF2 is expressed in early Myf5+/Pdgfrα+ brown progenitors as well as in Pdgfrα+ beige precursors from subQ WAT, serving as potential markers of these progenitors (55). However, classical BAT of EBF2 KO mouse shows normal levels of pan-adipocyte markers, PPARγ, adiponectin, and Fabp4, but loss of BAT-specific Ucp1, PRDM16, and Cidea expression, demonstrating that EBF2 is not required for general adipogenic process but specifically regulates BAT-specific gene expression (54, 55). Interestingly, a recent study showed that EBF2 forms a ribonucleoprotein complex with a long non-coding RNA (IncRNA) termed brown fat lncRNA 1 (Blnc1), which is transcriptionally regulated by EBF2 during brown adipogenesis, to promote adipogenesis in brown adipocytes (56).

## KLF11, IRF4, and ZFP516

As aforementioned and reviewed in Ref. (25, 57), several KLF-family proteins play important roles in the common adipogenic differentiation of BAT and WAT. Notably, KLF11 was recently identified as an activator of beige cell differentiation of human adipose-derived stem cells (58). KLF11 is a direct target of PPARγ and activates the expression of beige-specific genes. Expression of interferon regulatory factor 4 (IRF4) is induced by cold in both BAT and WAT and overexpression of IRF4 in BAT and WAT leads to enhanced thermogenesis and resistance to HFD-induced obesity (59). Conversely, specific deletion of IRF4 in Ucp1+ cells (brown and beige cells) causes a reduction in energy expenditure and thermogenesis as well a block in beige cell formation in subQ WAT. Interestingly, PGC-1α interacts with IRF4 and this interaction appeared to be crucial for activation of Ucp1 expression. A search for transcription factors that directly activate Ucp1 led to an identification of Zfp516 containing ten C2H2 zinc finger protein (60). Ectopic expression or genetic deletion of Zfp516 results in browning of WAT or loss of classical BAT development. Though the exact mechanisms of Zfp516 are not clear, it interacts with PRDM16; however, since PRDM16 is dispensable for classical BAT development, how Zfp516 regulates BAT development remains unresolved.

## Inhibitors of Brown and Beige Cell Differentiation: Rb Family Proteins and MRTFA

Retinoblastoma susceptibility (Rb) family proteins, Rb and p107, have important roles in determining white versus brown adipocyte differentiation (61, 62). Deletion of *Rb* in MEFs or embryonic stem (ES) cells results in brown fat differentiation (Ucp1+) upon adipogenic stimulation while control cells give rise to white adipocytes (61). Consistent with this, mesenchymal progenitor-specific Rb KO embryos show a significant increase in classical BAT mass (63). Intriguingly, while Rb is required for white adipocyte differentiation *in vitro* (64, 65), adipose-specific KO of Rb (66) or inactivation of Rb via SV40 T antigen in WAT (67) results in browning of WAT. Expression of p107 is abundant in the SVF from Vis WAT, lower in subQ WAT, and absent in BAT (68). Mature white adipocytes from any WAT depots do not express p107. Deletion of p107 in a congenic Balb/c background leads to an impairment of WAT development but not BAT, and causes extensive browning in various WAT depots (62). β-adrenergic stimulation reduces p107 expression in SVF and induces beige fat differentiation (68). Thus, Rb family proteins likely function as a negative regulator of beige cell differentiation. Similarly, genetic ablation of myocardin-related transcription factor A (MRTFA) results in browning of WAT depots without affecting BAT mass and function (69). MRTFA KO mice are protected from HFD-induced obesity and insulin resistance, demonstrating that MRTFA is a negative regulator of beige cell formation.

## Regulation of Brown and Beige Cell by microRNAs

Recent studies have identified several miRNAs that specifically target the expression of critical brown or beige transcription factors described above. Accordingly, many miRNAs are expressed in BAT- or WAT-specific manner. One such miRNA, miR-193b-365, is activated by PRDM16 and is required for brown adipogenesis (70) (Figure 1). Forced expression of miR-193b-365 in myoblasts blocks myogenesis and upon adipogenic stimulation, induces brown fat differentiation. In contrast, miR-133a represses PRDM16 expression and inhibits brown fat differentiation by targeting the 3′-UTR of PRDM16 transcripts (71, 72). Genetic inactivation of miR-133a has no effects on BAT development but increases beige cell development in WAT depots, which results in improved thermogenesis and glucose and insulin sensitivity. TGFβ, a potent inhibitor of adipogenesis (73), increases the expression of miR-155 and inhibits adipogenesis (74). Inhibition of adipogenesis by miR-155 overexpression suppresses CEBPβ expression, while CEBPβ represses miR-155 expression, forming a double negative loop in brown adipogenesis (Figure 1). Ectopic expression of miR-155 in the mouse reduces BAT size and function while miR-155 KO mice show improved BAT thermogenesis and enhanced beige cell formation in WAT. Expression of miR-196a is induced in subQ WAT following cold or β3-agonist stimulation and is required for Ucp1 expression (75). miR-196a represses the expression Hoxc8, homeobox c8, which is highly expressed in white fat cells and inhibits brown fat differentiation. Adipose-specific expression of miR-196a results in enhanced browning of WAT and protects mice from HFD-induced obesity and insulin resistance. Intriguingly, it was found that Hoxc8 represses CEBPβ expression by recruiting histone deacetylase, HDAC3. Thus, miR-196a regulates the expression of CEBPβ through Hoxc8 (Figure 2). miR-26a and miR-26b also have positive effects on converting human preadipocytes into beige cells (76). This is mediated by repressing ADAM17, ADAM metallopeptidase domain 17, expression and knockdown of ADAM17 recapitulates the increased beige adipogenesis. On the flip side, miR-27a/b negatively regulates multiple critical regulators of brown and beige adipogenesis, such as PRDM16, PPARγ, and PGC-1β, and its expression is repressed by cold exposure in BAT and subQ WAT (77).

In this short review, we highlighted the roles of the transcriptional regulators and miRNAs on brown and beige cell differentiation and function. While we have learned a great deal about brown and beige fat cells, there are still many unanswered questions. Recent studies suggest that cold- or β-adrenergic-stimulated induction of beige cells in WAT is transient and reversible (i.e., reversible transdifferentiation between mature white fat and beige cells) (17, 18). To achieve this plasticity, mature white fat and beige cells must have mechanisms to tightly and reciprocally regulate many of the beige-specific transcriptional regulators and miRNAs during this reversible process (Figure 2). As exemplified by the CEBPβ-miR-155 regulatory loop (75), delineating the intricate details of the interdependence and cross-regulation of the transcription factor and miRNA networks will provide a deeper understanding of brown and beige fat differentiation and facilitate the development of brown or beige cell-based therapy.

## Conflict of Interest Statement

The authors declare that the research was conducted in the absence of any commercial or financial relationships that could be construed as a potential conflict of interest.
